# Compost and biochar alter mycorrhization, tomato root exudation, and development of *Fusarium oxysporum* f. sp. *lycopersici*

**DOI:** 10.3389/fpls.2015.00529

**Published:** 2015-07-10

**Authors:** Adnan Akhter, Karin Hage-Ahmed, Gerhard Soja, Siegrid Steinkellner

**Affiliations:** ^1^Division of Plant Protection, Department of Crop Sciences, University of Natural Resources and Life Sciences ViennaTulln, Austria; ^2^Department of Health and Environment, Austrian Institute of TechnologyTulln, Austria

**Keywords:** *Fusarium oxysporum*, compost, biochar, mycorrhizal fungi, tomato root exudates, disease suppression

## Abstract

Soil amendments like compost and biochar are known to affect soil properties, plant growth as well as soil borne plant pathogens. Complex interactions based on microbial activity and abiotic characteristics are supposed to be responsible for suppressive properties of certain substrates, however, the specific mechanisms of action are still widely unknown. In the present study, the main focus was on the development of the soil borne pathogen, *Fusarium oxysporum* f.sp. *lycopersici* (*Fol*) in tomato (*Solanum lycopersicum* L.) and changes in root exudates of tomato plants grown in different soil substrate compositions, such as compost (Comp) alone at application rate of 20% (v/v), and in combination with wood biochar (WB; made from beech wood chips) or green waste biochar (GWB; made from garden waste residues) at application rate of 3% (v/v), and/or with additional arbuscular mycorrhizal fungi (AMF). The association of GWB and AMF had a positive effect on tomato plants growth unlike to the plants grown in WB containing a soil substrate. The AMF root colonization was not enhanced by the addition of WB or GWB in the soil substrate, though a bio-protective effect of mycorrhization was evident in both biochar amended treatments against *Fol*. Compost and biochars altered root exudates differently, which is evident from variable response of *in vitro* growth and development of *Fol*. The microconidia germination was highest in root exudates from plants grown in the soil containing compost and GWB, whereas root exudates of plants from a substrate containing WB suppressed the mycelial growth and development of *Fol*. In conclusion, the plant growth response and disease suppression in biochar containing substrates with additional AMF was affected by the feedstock type. Moreover, application of compost and biochars in the soil influence the quality and composition of root exudates with respect to their effects on soil-dwelling fungi.

## Introduction

Plant diseases are always a danger to world's food security and often difficult to control with modern agricultural practices like the use of disease resistant cultivars and synthetic pesticides. The increasing incidence of fungicide resistance and the failure of host resistance against pathogens are among the driving forces to develop new disease management strategies (Mcdonald and Linde, 2002). The use of organic matter inputs such as biochar and compost might be a promising approach, as their suppressive effect has been shown for a wide range of soil borne diseases (Coventry et al., 2005; Noble and Coventry, 2005).

Biochar is a carbon rich product of a heating process in an oxygen depleted environment known as pyrolysis (Sohi et al., 2010; Elad et al., 2011; Sparks, 2011). The type of organic material (e.g., agricultural crop residues, forestry waste, wood chips, etc.) and heating temperature used for the production of biochar determine its nutrient contents and physicochemical properties (Antal and Grønli, 2003; Gaskin et al., 2008). A biochar addition to the soil may improve the physicochemical properties of soil like bulk density, water holding capacity, nutrient retention, soil pH, and cation exchange capacity resulting into beneficial effects on plant growth (Glaser et al., 2002; Steiner et al., 2008; Atkinson et al., 2010). Biochar is very stable in soil with a half-life ranging up to thousands of years (Zimmerman, 2010). Recently, it has been reported that soil with biochar can trigger systemic plant defense and suppress the disease severity of foliar pathogens like *Botrytis cinerea* and *Leveillula taurica* in tomato and pepper respectively (Elad et al., 2010) and in strawberry plants against *Botrytis cinerea*, *Colletotrichum acutatum* and *Podosphaera aphanis* (Harel et al., 2012). Elmer and Pignatello (2011) reported the reduction in root lesions due to *Fusarium oxysporum* f. sp. *asparagi* and *F. proliferatum* in the presence of biochar in the soil substrate.

Another soil amendment with known suppressive effects is compost. Compost is a product of organic residues produced by aerobic biological decomposition (biodegradation process). Composts are known to suppress a wide variety of diseases caused by various soil borne pathogens, including *Fusarium* species (Bonanomi et al., 2007). The application of composted organic wastes can suppress the tomato wilt caused by *F. oxysporum* f.sp. *lycopersici* (Borrero et al., 2004). This could be due to an enhanced competition and antagonism by the soil biota associated with increased microbial activity in the soil (Pugliese et al., 2011). Arabidopsis plants exhibit a high level of resistance to *Botrytis cinerea* when grown in olive marc compost (Segarra et al., 2013). The reduction of foliar diseases due to the application of compost is attributed to salicylic acid signaling. A combination of biochar with compost has positive effects on plant growth (Schulz and Glaser, 2012) and this phenomenon may also be attributed to a stimulated activity of beneficial microorganisms in the rhizosphere as an increase in the microbial population and reproduction rate has been reported for biochar amended soils (Steiner et al., 2004; Graber et al., 2010; Jin, 2010).

Arbuscular mycorrhizal fungi (AMF) comprise a large portion of soil and rhizosphere microbiota. AMF share symbiotic associations with many important plant species and affect soil borne pathogens (Whipps, 2004) as well as foliar necrotrophic pathogens like *Alternaria solani* (Fritz et al., 2006). There is a continuous communication between mycorrhizal hyphal networks and roots of the host plant affecting the host metabolism (Smith and Read, 2008; Johnson and Gilbert, 2015). It is already known that colonization by AMF induces changes in the plant root exudates. Root exudates from mycorrhizal and non mycorrhizal plants have different effects on soil borne pathogens (Scheffknecht et al., 2006). The application of AMF with biochar may contribute to the nutrient uptake (Hammer et al., 2014) but the effect of biochar on AMF could be either positive or negative depending on soil characteristics and other soil microorganisms.

Plant roots exude a diverse range of compounds to facilitate nutrient uptake, communicate with rhizosphere microorganisms and to cope with plant pathogens (Bais et al., 2004; Haichar et al., 2014). Organic acids like citric and succinic acid are the major sources of carbon along with sugars like fructose and glucose in tomato root exudates (Kamilova et al., 2006). The composition of root exudates has a strong influence on the establishment and composition of the root-associated microbial community (Walker et al., 2003). The interactions between soil borne plant pathogenic fungi and their host plants are mediated via root exudates to a substantial extent. The quality and quantity of root exudates depends on the plant species and growth conditions (Curl and Truelove, 1986; Jones et al., 2004). It has been reported by Steinkellner et al. (2005, 2009) that the microconidia germination of the tomato pathogen *F. oxysporum* was stimulated by root exudates of tomato. However, little is known about the root exudates exuded by the plants as a consequence of additional organic amendments in the plant growth medium. The information is scarce regarding the possible effects of biochar and other organic amendments like compost application on the pattern of plant root exudation.

In our study we are focusing on the economically important pathosystem tomato (*Solanum lycopersicum* L.), and *F. oxysporum* f.sp *lycopersici (Fol)*. Tomato is the most important vegetable crop grown and consumed globally. In terms of the monetary value tomato is ranked at 8th place among all the food and agricultural products produced in the world (FAOSTAT, 2012). The tomato wilt caused by *Fol* pose a serious threat to tomato production all over the world causing huge economic losses both in the greenhouse and in the field (McGovern, 2015). *Fol* is saprophytic in nature and can survive in the form of mycelium and chlamydospores in the soil and plant debris for a longer period of time (Agrios, 1997). During its life cycle *Fol* produces three type of spores (i) microconidia (ii) macroconidia and (iii) chlamydospores with a different infection potential (De-Cal et al., 1997). The main objectives of the study were (I) to assess the changes in root exudation of mycorrhizal and non mycorrhizal tomato plants in response to the application of compost and biochar by evaluating the effects of root exudates on the *in vitro* growth and the development of *Fol* and II) to determine the effect of compost and biochar in combination with AMF on plant growth and on disease suppression. As *Fol* is a soil borne pathogen, a reduction in the disease severity and incidence partially may be credited to the alteration of root exudates composition in response to compost and biochar, apart from differences in plant defense mechanisms.

## Materials and methods

### Fungal culture

*F. oxysporum* f. sp. *lycopersici* (isolate 007) was cultivated for 2–3 weeks at 24°C in darkness on Czapek Dox (CZD) agar (Duchefa Biochemie, Haarlem, The Netherlands). The microconidia were harvested by flooding the *Fusarium* culture with autoclaved, distilled water and gently rubbing with a Drigalski spatula. The conidial suspension was filtered through three layers of fleece filters (150 μm) and a final concentration was determined and adjusted at 1 × 10^7^ microconidia/ml with a haemocytometer (Steinkellner et al., 2008).

For AMF inoculation, Symbivit (Symbivit®, Zivojin Rilakovic, Guntramsdorf, Austria) was utilized as a commercially available inoculum. This AMF inoculum holds at least 80,000 spores/liter and includes six different species of AMF (*Claroideoglomus etunicatum, Glomus microagregatum*, R*hizophagus intraradices, Glomus claroideum, Funneliformis mosseae*, and *Funneliformis geosporum*) (Hage-Ahmed et al., 2013).

### Soil preparation

A sterilized mixture of sand (Quarzsand 0–3 mm, Quarzwerke Österreich GmbH, Melk, Austria), soil (Aussaaterde, Gramoflor GmbH & Co. KG, Vechta, Germany), and expanded clay (Liapor fit 1–4 mm, Lias Österreich GmbH, Fehring, Austria) (1:1:1, v/v/v) was used as basic material to make combinations of compost (Comp) at 20% and/or biochar at 3% (v/v) for the plant cultivation. The compost originated from the municipal compost works in Klosterneuburg with a compost quality A+, categorized according to the Austrian compost regulation (BGBl. II Nr. 929/2001). Two types of biochar depending on the feedstock were used in this study (i) wood biochar (WB) made from beech wood chips and green waste biochar (GWB) from garden waste residues at pyrolysis temperature of 500°C. Both of the biochars in this study were the same as utilized and analyzed previously by Frišták et al. (2015). GWB has a comparatively higher cation exchange capacity, surface area and nitrogen contents than WB. Table 1 summarizes the characteristics of compost, WB and GWB. GWB was sieved through 2 mm sieve before use (Kloss et al., 2014).

**Table 1:** **Physicochemical parameters of compost, wood biochar and green waste biochar**.

**Parameters measured**	**Compost**	**Wood biochar**	**Green waste biochar**
pH	7.10^a^	8.78^b^	9.03^b^
Carbon (%)	27.00	80.30	79.78
Nitrogen (%)	2.20	0.40	0.65
Conductivity (mS/cm)	1.40	0.54	1.67
Ash contents (%)	----	15.20	19.30
Cadmium (mg/kg)	0.14	<2.00	<2.00
Copper (mg/kg)	86.00	16.00	21.00
Zinc (mg/kg)	321.00	93.00	95.00
Density (kg/L)	0.77	0.36	0.34
CEC(mmol 100/mL)	----	9.83	12.85

a pH in CaCl_2._

b pH was measured in de-ionized water.

---- Parameters were not analyzed.

The following treatments were used in the experimental setup: (i) Comp (ii) Comp+WB (iii) Comp+GWB, with (+AMF) and/or without AMF (−AMF). The treatments were either free from *Fol* (–Fol) or inoculated with *Fol* (+Fol). Each treatment comprised 5 replicates and each replicate consisted of a pot with one tomato plant. The experiments were conducted thrice.

### Plant material and root exudate extraction

The tomato (*Solanum lycopersicum* L. cv. Kremser Perle) seeds were surface-sterilized with 50% household bleach (3.8% NaOCl) by soaking for 10 min and washed three times with autoclaved distilled water afterwards. The tomato seeds were transferred to pots filled with sterilized perlite and incubated in a growth chamber (Rumed, Rubarth Apparate GmbH, Germany) with a 16-h light/8-h dark photoperiod (light intensity 296 μmol/m^2^/s) at 24°C. Tap water was used for watering the perlite. The tomato seedlings were grown for 4 weeks before transplanting into the prepared potting mixes. For *Fol* inoculation, the tomato seedlings were gently removed from the perlite. The roots of the tomato plantlets were clipped and dipped for 5 min in the conidial suspension containing 1 × 10^5^ microconidia/ml. The AMF inoculation was done by placing 4 ml of inoculum into the planting hole before the transplantation. The plants were grown in the greenhouse with a random design under long day conditions for 6 weeks and watered with tap water regularly to maintain the optimum moisture conditions (Steinkellner et al., 2005). Afterwards the plants were harvested by gently uprooting from the substrate and washing the roots under running tap water. In each treatment 5 plants were used for the extraction of root exudates. The root exudates were extracted in acetate buffer (25 mM, pH = 5.5) for 6 h as described by Hage-Ahmed et al. (2013a). The final concentration of the root exudates was adjusted with acetate buffer to 20 ml/g of root fresh weight. The exudates were filtered through 0.22 μm sterile filters (Steriflip, Millipore, Bedford, USA) and stored at −80°C till further use.

### Agronomic and physiological parameters

After the root exudate extraction roots and shoots were separated and their fresh weight was calculated. The phenological development stage (BBCH-scale) was recorded according to Feller et al. (1995). The maximum photochemical efficiency of photosystem II [PSII (Fv/Fm)] was calculated by measuring the fast kinetics of chlorophyll fluorescence using the chlorophyll fluorimeter, Handy PEA (Hansatech Instruments Ltd., Norfolk, UK). The leaves were dark adapted for 30 min before the measurement. Chlorophyll contents of the leaves were determined by using a portable Chlorophyll meter (SPAD 502 Plus, Minolta, Japan), 2 days before harvesting of the plants.

### Disease assessment and AMF colonization

The confirmation of *Fol* infection was done by a visual observation and by incubating a surface sterilized piece of the shoot base of 0.5 cm in length on media plates of potato dextrose agar amended with streptomycin (10 mg/l) at 24°C in the dark (Steinkellner et al., 2012). Disease incidence was calculated as percent of infected plants to the total number of plants of the corresponding treatment according to the following formula:

$$\text{Disease incidence}=\frac{\text{Number of infected plants}}{\text{Total number of plants}}\text{ x }100$$

For the disease severity assessment, each plant stem was split open and the % length of discolored vascular tissue to the total length of the stem was calculated (Hage-Ahmed et al., 2013). Based on the infected stem length (%) plants were rated on a scale of 1–5 categories modified from Wellman (1939) (c1 = 1–5%, c2 = 5–15%, c3 = 15–35%, c4 = 35–67.5%, c5 = 67.5–100% of stem vascular tissue discoloration). The disease severity was calculated for each batch of 5 plants separately by the following formula:

$$\text{Disease severity}=\frac{5\text{ x }(\text{nc}1+2\text{nc}2+5\text{nc}3+10\text{nc}4+20\text{nc}5)}{\text{n infected plants}}$$

For the assessment of AMF colonization root segments of 2 cm in length, starting 2 cm from base of the shoot were prepared by clearing them in 10% KOH solution at 90°C for 3–4 min followed by three washings with tap water. Afterwards the roots were stained with a 5% ink-vinegar solution (Vierheilig et al., 1998) at 90°C for 3 min and rinsed 3 times with tap water. The AMF root colonization rate was determined as described by McGonigle et al. (1990).

### Fungal growth assay (microconidia germination and mycelial growth assessment in root exudates)

The *Fol* spore germination assays were done in 96-well plates (NUNCLONTM D Surface, F96 MicroWellTM Plates, NUNCTM Brand Products, Roskilde, Denmark). For each exudate three replicates were made. Each well comprised of 175 μl of root exudates and 35 μl of a conidial spore suspension (1 × 10^7^ microconida/ml). The plates were incubated at 24°C in the dark on a rotary shaker at 200 rpm for 20 h. The germination rate (%) was determined microscopically after 20 h by counting 200 spores. For each 96-well plate CZD broth was used as a positive control and acetate buffer as a negative control. The plates for the mycelial growth assay were prepared as described above. The mycelial growth was determined by measuring the optical density (600 nm) with a spectrophotometer (FLUOstar Omega, BMG LABTECH GmbH, Ortenberg, Germany) after every 24 h for 5 consecutive days (Steinkellner and Mammerler, 2007).

### Statistical analysis

The data analysis was performed with the PASW Statistics 18 (Version 18.0.0, IBM, Armonk, NY, USA) software. The data were analyzed for homogeneity of variance (Levene's test). Data were transformed for the AMF root colonization rate and for the mycelial growth at 20 h. Afterwards the treatments were subjected to a Two-Way analysis of variance (ANOVA) and means were separated with the Tukey's test (*P* = 0.05).

## Results

### Estimation of growth parameters

**Figure 1:**
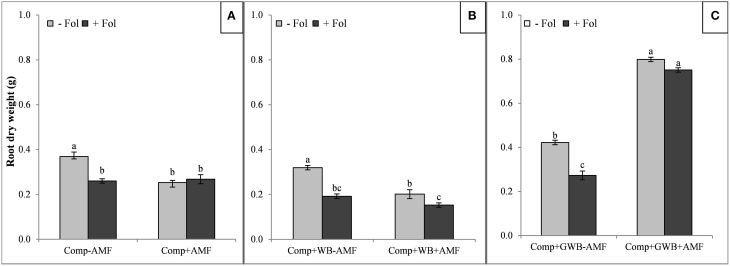
**Effect of AMF and** ***F. oxysporum*** **f.sp.** ***lycopersici*** **on root dry weight of tomato plants grown in different soil substrates, (A) comprising of compost (Comp) inoculated (+Fol) or un-inoculated (−Fol), with AMF (+AMF) or without AMF (−AMF) (B) combination of compost and wood biochar (Comp+WB), inoculated (+Fol) or un-inoculated (−Fol), with AMF (+AMF) or without AMF (-AMF) (C) combination of compost and GWB (Comp+GWB) was utilized under the same settings as described for (A,B)**. Data were recorded 6 weeks after transplanting. Each bar represent mean ± SE, *n* = 15, bars with different letters indicate significant differences according to Tukey's test (*P* < 0.05).

**Figure 2:**
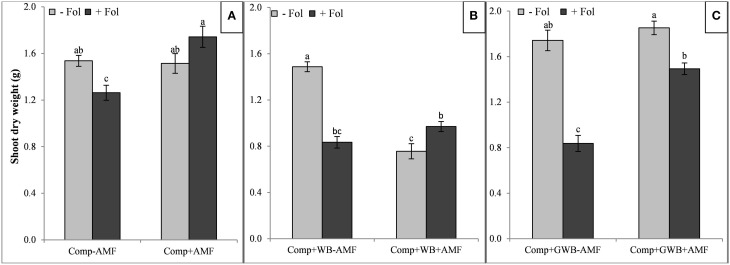
**Effect of AMF and** ***F. oxysporum*** **f.sp.** ***lycopersici*** **on shoot dry weight of tomato plants grown in different soil substrates, (A) comprising of compost (Comp) inoculated (+Fol) or un-inoculated (−Fol), with AMF (+AMF) or without AMF (−AMF) (B) combination of compost and wood biochar (Comp+WB), inoculated (+Fol) or un-inoculated (−Fol), with AMF (+AMF) or without AMF (−AMF) (C) combination of compost and GWB (Comp+GWB) was utilized under the same settings as described for (A,B)**. Data were recorded 6 weeks after transplanting. Each bar represent mean ± SE, *n* = 15, bars with different letters indicate significant differences according to Tukey's test (*P* < 0.05).

The root and shoot dry mass of tomato plants grown in each soil substrate composition is shown in Figures 1, 2, respectively. There was a reduction of the root dry weight of *Fol* (+Fol) inoculated plants grown in compost alone and in combination with WB in the presence (+AMF) or absence of AMF (−AMF) (Figures 1A,B). The minimum root dry weight (0.15 g) was recorded in plants co-inoculated with *Fol* and AMF grown in WB containing soil substrate (Comp+WB+AMF+Fol) (Figure 1B). A higher biomass (root dry weight 0.80 g) of AMF colonized plant roots was recorded in plants grown in the “Comp+GWB+AMF” treatment and the root dry weight was sustained by plants even under *Fol* stress (Comp+GWB+AMF+Fol; 0.75 g) (Figure 1C). There was 2-and 3-fold increase in root dry weight in the “Comp+GWB+AMF” and “Comp+GWB+AMF+Fol” treatments respectively as compared to their -AMF complements. The interactive effect of *Fol* and AMF was observed for soil substrates comprising of compost alone (Comp) [*F*_(1, 56)_ = 29.83, *P* < 0.001] and in combination with WB (Comp+WB) [*F*_(1, 56)_ = 16.27, < 0.001] as well as for GWB containing substrate (Comp+GWB) [*F*_(1, 56)_ = 17.42, < 0.001].

The shoot dry weight was lowest in the “Comp+WB” treatment with *Fol* and/or AMF (Figure 2B) and highest in the “Comp+AMF+Fol,” (Figure 2A) “Comp+GWB–AMF” and the “Comp+GWB+AMF” treatment (Figure 2C). The “*Fol*” inoculation reduced the shoot dry weight except in combination with “Comp+AMF.” The maximum shoot dry weight (1.85 g) was recorded in “Comp+GWB+AMF” and the minimum (0.76 g) in “Comp+WB+AMF” (Figures 2B,C respectively). However, in the absence of *Fol*, there was no significant difference in the shoot dry weight of tomato plants grown in “Comp+AMF” and “Comp+GWB+AMF” treatments compared to their respective −AMF compliments (Figures 2A,C). The shoot dry weight was also affected by the interactive effect of AMF × *Fol* in each soil substrate composition [“Comp,” “Comp+WB” and “Comp+GWB”; *F*_(1, 56)_ = 11.64, *P* < 0.001; *F*_(1, 56)_ = 70.49, *P* < 0.001, *F*_(1, 56)_ = 15.46, *P* < 0.001 respectively].

The results of the Two-Way ANOVA revealed that AMF and *Fol* inoculation and their interaction significantly affected the root and shoot dry weight except in case of the soil substrate containing compost alone where *Fol* had no significant effect on the shoot dry weight (Table 2).

**Table 2:** **Results from Two-Way ANOVA presented as degrees of freedom (*****df*****), F values and level of significance of the main factors (AMF and** ***Fol*****) and their interaction (AMF × Fol) on tested variables of tomato plants grown in different soil substrate compositions (Comp, Comp+WB and Comp+GWB)**.

**Treatments**			**Root dry weight**		**Shoot dry weight**			**Chlorophyll contents**		**Maximum Photochemical efficiency (Fv/Fm)**	
		***df***	***F***	***P***	***F***	***P***	***df***	***F***	***P***	***F***	***P***
Comp	AMF	1	19.92	**<0.001**	9.64	**0.003**	1	93.37	**<0.001**	0.35	0.559
	*Fol*	1	14.57	**<0.001**	0.01	0.759	1	50.15	**<0.001**	5.87	**0.025**
	AMF × *Fol*	1	29.83	**<0.001**	11.64	**<0.001**	1	2.69	**<0.001**	0.19	0.193
Comp+WB	AMF	1	54.37	**<0.001**	32.84	**<0.001**	1	8.33	**0.009**	0.00	0.958
	*Fol*	1	70.25	**<0.001**	18.17	**<0.001**	1	35.67	**<0.001**	31.58	**<0.001**
	AMF × *Fol*	1	16.27	**<0.001**	70.48	**<0.001**	1	3.67	0.070	3.31	0.084
Comp+GWB	AMF	1	1376.57	**<0.001**	30.40	**<0.001**	1	41.76	**<0.001**	0.04	0.841
	*Fol*	1	69.20	**<0.001**	82.64	**<0.001**	1	101.95	**<0.001**	5.24	**0.033**
	AMF × *Fol*	1	17.42	**<0.001**	15.46	**<0.001**	1	87.54	**<0.001**	2.97	0.100
	Error *df*	**56**					**20**				

Significant P values (P < 0.05) are in bold.

The chlorophyll contents decreased significantly in *Fol* inoculated plants; however AMF incorporation in the soil substrate had a positive influence on chlorophyll contents (Table 3). There was significant [*F*_(1, 20)_ = 87.54, *P* < 0.001] interactive effect of AMF × *Fol* in the GWB containing soil substrate unlike treatments “Comp” and “Comp+WB” (Table 2). Tomato plants grown in “Comp+GWB” produced the highest chlorophyll contents (41.82 ± 0.59) while the lowest level (36.22 ± 0.49) was recorded in the treatment “Comp+WB+Fol.”

**Table 3:** **Effect of AMF and** ***F. oxysporum*** **f.sp.** ***lycopersici*** **on chlorophyll contents and photochemical efficiency of PSII of tomato plants grown in different soil substrate compositions**.

**Treatments**		**Chlorophyll contents (spad value)**	**Maximum photochemical efficiency (Fv/Fm)**
Comp-Fol	−AMF	38.40 ± 0.57^b^	0.79 ± 0.01^a^
	+AMF	41.10 ± 0.53^a^	0.79 ± 0.01^a^
Comp+Fol	−AMF	37.10 ± 0.75^c^	0.78 ± 0.02^a^
	+AMF	39.02 ± 0.46^b^	0.77 ± 0.02^a^
Comp+WB-Fol	−AMF	38.51 ± 0.55^ab^	0.77 ± 0.02^c^
	+AMF	38.80 ± 0.95^a^	0.78 ± 0.01^bc^
Comp+WB+Fol	−AMF	36.22 ± 0.49^c^	0.82 ± 0.01^a^
	+AMF	37.62 ± 0.77^b^	0.81 ± 0.01^ab^
Comp+GWB-Fol	−AMF	41.82 ± 0.59^a^	0.81 ± 0.01^a^
	+AMF	41.10 ± 0.48^a^	0.79 ± 0.01^ab^
Comp+GWB+Fol	−AMF	37.00 ± 0.89^b^	0.77 ± 0.04^b^
	+AMF	40.92 ± 0.31^a^	0.79 ± 0.01^ab^

Data were mean values ± SD (n = 6) followed by different letters indicate significant differences according to Tukey's test (P < 0.05).

There was a significant reduction in the photosystem II (PSII) efficiency (Fv/Fm) of *Fol* inoculated tomato plants grown in treatments “Comp+AMF+Fol” (0.77 ± 0.02) and “Comp+GWB+Fol” (0.77 ± 0.04) [*F*_(1, 20)_ = 5.87, *P* = 0.025; *F*_(1, 20)_ = 5.24, *P* = 0.033 respectively] whereas an increase was observed in the plants grown in the WB containing substrate (Comp+WB+Fol; 0.82 ± 0.01) [*F*_(1, 20)_ = 31.58, *P* < 0.001] (Tables 2, 3). The treatment “Comp” and “Comp+WB” in combination with AMF had a non-significant impact on the PSII efficiency as compared to −AMF plants. However, in “Comp+GWB+Fol” the incorporation of AMF had a positive effect on the PSII efficiency.

### Disease incidence and severity assessment

The assessment of *Fol* incidence and severity on tomato plants was done 6 weeks after transplanting. There was reduction in disease incidence of *Fol* in tomato plants grown in the compost associations with GWB and WB in the presence of AMF (Table 4). The disease incidence was lowest (40%) in the AMF colonized plants grown in the GWB containing soil (Comp+GWB+AMF+Fol). In contrast the incorporation of AMF in soil substrate containing only compost disease incidence was increased from 33.33% (Comp+Fol) to 53.33% in the “Comp+AMF+Fol” treatment. The main factors treatment and AMF had significant [*F*_(2, 12)_ = 31.59, *P* < 0.001; *F*_(1, 12)_ = 15.45, *P* < 0.001 respectively] effects on the disease severity along with the interactive effect of treatment × AMF [F_(2, 12)_ = 20.87, *P* < 0.001]. The incorporation of AMF had a positive influence on the disease severity reduction in the WB and GWB containing treatments. There was a 51.48 and 35.94% reduction in disease severity in the “Comp+WB+AMF+Fol” and “Comp+GWB+AMF+Fol” treatments, respectively, as compared to their non-AMF counterparts. However, in the “Comp+AMF+Fol” treatment the disease severity was significantly increased (8.53 ± 1.29) due to the presence of AMF compared to the -AMF treatment (Comp+Fol; 3.87 ± 1.63). However, the differences in disease severity were not significant between the WB and GWB containing treatments.

**Table 4:** **Effect of different soil substrates [compost (Comp), combination of compost with wood biochar (Comp+WB) and with green waste biochar (Comp+GWB) inoculated with** ***Fol*** **(+Fol), with (+AMF) or without AMF (−AMF)] on the disease incidence and severity by** ***F. oxysporum*** **f.sp.** ***lycopersici*** **on tomato plants, 6 weeks after inoculation**.

**Treatments**	**Comp+Fol**		**Comp+WB+Fol**		**Comp+GWB+Fol**	
	**−AMF**	**+AMF**	**−AMF**	**+AMF**	**−AMF**	**+AMF**
Disease incidence (%)^1^	33.33	53.33	53.33	46.67	46.67	40.00
Disease Severity^*^	3.87 ± 1.63^c^	8.53 ± 1.29^b^	17.87 ± 3.35^a^	8.67 ± 1.53^b^	17.78 ± 1.92^a^	11.39 ± 1.27^b^

1 Data were calculated for total number of plants in each treatment.

* Data were mean values ± SD, followed by different letters indicate significant differences according to Tukey's test (P < 0.05).

### AMF colonization rate

**Figure 3:**
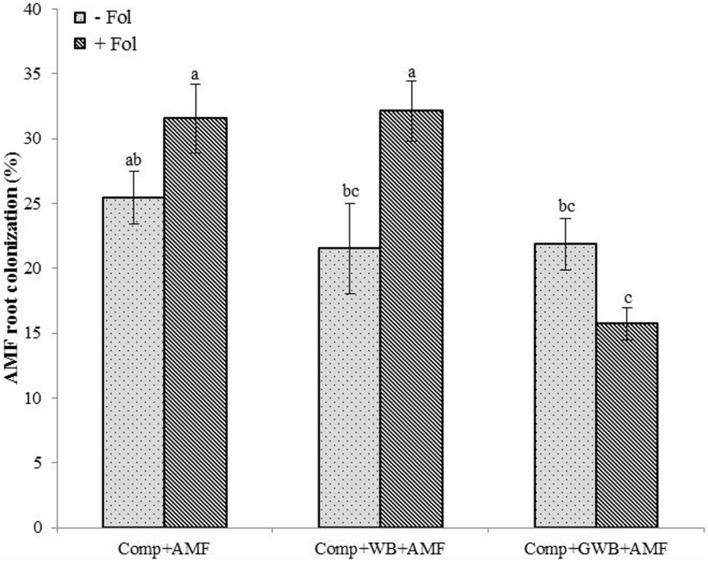
**Effect of different soil substrate compositions comprising of compost (Comp), and combination of compost with WB (Comp+WB) and with GWB (Comp+GWB), un-inoculated (−Fol) and inoculated (+Fol) on tomato plants root colonization by AMF**. Each bar represent mean ± SE, *n* = 15, bars with different letters indicate significant differences according to Tukey's test (*P* < 0.05).

Depending on the soil substrate composition and *Fol* inoculation, the tomato root colonization by AMF ranged between 15.71 and 32.14% (Figure 3). The AMF colonization rate was influenced by the significant main effect of treatment (different soil substrate compositions) [*F*_(2, 84)_ = 12.06, *P* < 0.001] and the interactive effect of treatment × *Fol* [*F*_(2, 84)_ = 10.52, *P* < 0.001] inoculation of tomato plants. The *Fol* inoculated tomato plants grown in the soil substrate consisting of compost alone (Comp+AMF+Fol) and in combination with WB (Comp+WB+AMF+Fol) resulted in an increased colonization of the tomato plant roots by AMF. Therefore, a maximum AMF root colonization (32.14 %) was observed in the plants grown in “Comp+AMF+Fol” followed by the “Comp+WB+AMF” (31.57%) treatment. The AMF root colonization was reduced in the plants from “Comp+WB+AMF,” “Comp+GWB+AMF” and from “Comp+GWB+AMF+Fol.”

### Effect of root exudates on *Fol* microconidia germination

**Figure 4:**
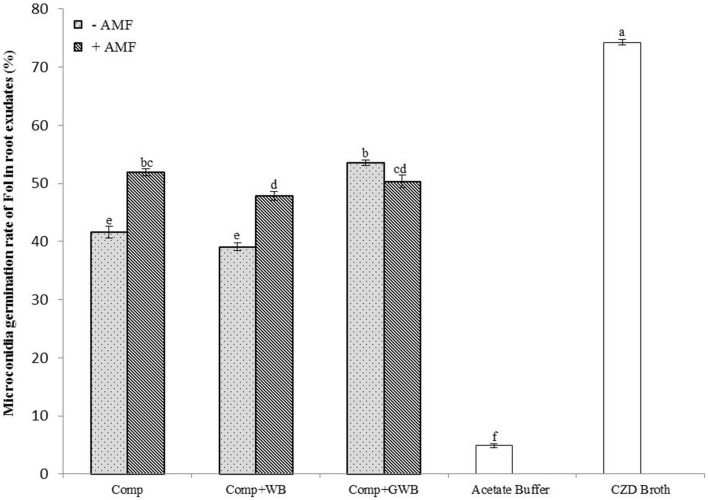
**Microconidia germination rate of** ***F. oxysporum*** **f.sp.** ***lycopersici*** **in root exudates from tomato plants grown in different soil substrates comprising of compost (Comp), combination of compost and wood biochar (Comp+WB) and with green waste biochar (Comp+GWB) with (+AMF) or without AMF (−AMF) after 20 h at 24°C**. Each bar represent mean ± SE (*n* = 9). Bars with no pattern represent microconidia germination in acetate buffer and Czapek dox medium (CZD). Different letters above bars indicate significant differences according to Tukey's test (*P* < 0.05).

The effect on the germination rate (%) of *Fol* microconidia was tested in root exudates from tomato plants grown in different soil substrates (Comp, Comp+WB and Comp+GWB) in the absence and presence of AMF (+AMF) (Figure 4). The pH of root exudates ranged between 5.79 and 6.32. The microconidia germination was highest (74.28%) in the CZD broth and lowest (4.89%) in the acetate buffer, positive and negative control respectively. There was a significant interactive effect of the treatment and AMF [*F*_(2, 48)_ = 63.53, *P* < 0.001] on microconidia germination. The *Fol* microconidia germination rate was significantly increased in the root exudates from AMF colonized plants grown in the “Comp+AMF” and “Comp+WB+AMF” treatments. However, the germination rate was reduced (50.33%) in root exudates from the “Comp+GWB+AMF” treatment as compared to its non-AMF counterpart (Comp+GWB; 53.5%). The minimum germination rate (39.06%) was detected in root exudates from −AMF plants grown in the WB containing treatment (Comp+WB).

### Effect of root exudates on *in vitro* mycelial growth and development

**Figure 5:**
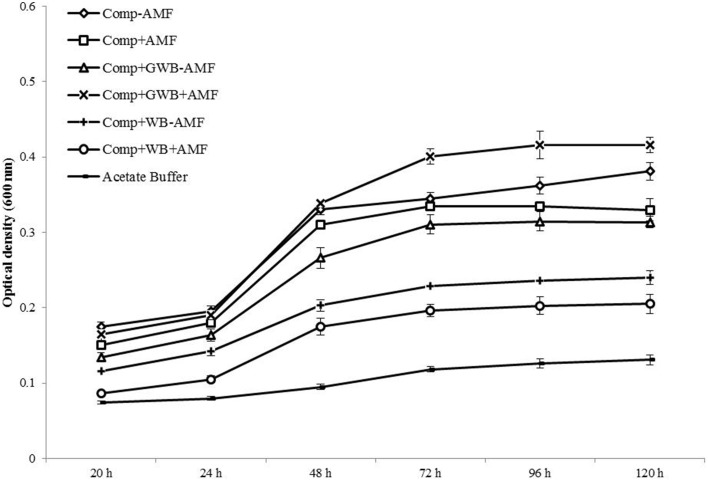
**Effect of root exudates of tomato plants on the mycelial growth of** ***F. oxysporum*** **f.sp.** ***lycopersici*****, monitored for a period of 5 days at 24°C after regular intervals**. Mycelial growth was assessed in root exudates extracted from plants grown in compost (Comp), and combination of compost with WB (Comp+WB) and with GWB (Comp+GWB), with (+AMF) or without AMF (−AMF).

The *Fol* mycelial growth and development in the root exudates was studied for a total of 120 h (Figure 5). CZD broth and acetate buffer served as control treatments. The fungal growth kept on increasing in CZD broth (1.97 ± 0.03) for the whole period but in acetate buffer (0.13 ± 0.02) the optical density remained in the lowest ranges. Tomato root exudates influenced the mycelial growth differently depending on the soil substrate composition and AMF colonization of the roots. After an incubation period of 120 h, a significant effect of the main factor treatment and an interactive effect of the treatment × AMF was observed [*F*_(2, 48)_ = 107.36, *P* < 0.001; *F*_(2, 48)_ = 30.42, *P* < 0.001 respectively]. Both WB and GWB containing treatments along with AMF altered the mycelial growth. The optical density was highest in root exudates from the “Comp+GWB+AMF” (0.42 ± 0.01) treatment whereas the root exudates from “Comp+WB+AMF” (0.21 ± 0.04) and “Comp+WB–AMF” (0.24 ± 0.03) had produced the lowest optical density. There was an enhanced stimulation (217% increase as compared to acetate buffer) of the mycelial growth in the root exudates from the “Comp+GWB+AMF” treatment followed by the treatment “Comp” (191% increase as compared to acetate buffer). However, the differences in the mycelial growth were not significant in plant root exudates taken from the “Comp+AMF” and “Comp+GWB–AMF” treatment.

## Discussion

Biochar application not only affects the crop yield (Kloss et al., 2014) but also has the ability to alter the plant response to disease stress (Graber et al., 2014). Previously, biochar has been reported to suppress fungal foliar and soil borne bacterial pathogens in tomato (Nerome et al., 2005; Elad et al., 2010, 2011), but there is no information available on the impact of biochar on the Fusarium wilt of tomato. In comparison, many studies document a suppressive effect of organic amendments like compost and organic wastes against *Fol* and other soil borne pathogens (Borrero et al., 2004; Bonanomi et al., 2007). This study is the first report on the effects of compost and biochar in combination with an AMF application on tomato plant growth and *Fol* disease development.

In our study, the amendment of WB and GWB to a substrate already containing compost affects plant growth only slightly in the absence of *Fol* and AMF. However, clear positive as well as negative effects on plant growth were evident under the influence of *Fol* and AMF. It is a well-documented fact that the feedstock and pyrolysis conditions determines the physicochemical properties of the biochar (Lehmann, 2007; Gaskin et al., 2008; Kloss et al., 2012), and the plants response with reference to growth promotion and disease suppression (Rajkovich et al., 2012; Jaiswal et al., 2014, 2015). Rajkovich et al. (2012) found either positive or negative changes in corn growth depended on the type of feedstock. Similarly, Chen et al. (2010) found a different growth response of sugarcane to biochar made from either bagasse or bio-solids.

In our study, the observed differences in plant response to AMF may also result from variations in the soil substrate characteristics (Clark and Zeto, 1996; Warnock et al., 2010), as each biochar type has the ability to modify the soil organic matter and nutrient status (Mukherjee and Zimmerman, 2013; Kloss et al., 2014). Recently, LeCroy et al. (2013) demonstrated that the combined treatment of apple wood saw dust biochar and mycorrhizal fungi with additional nitrogen fertilizer decreased aboveground sorghum biomass, even though biochar had a positive effect on the AMF root colonization in a greenhouse experiment. Moreover, they also noted a reduction in below ground sorghum biomass as well. We also observed a growth suppression of mycorrhizal plants in the WB treatment already containing compost as an additional source of nitrogen. This could be due to the carbon drainage to the fungal symbionts or a reduced availability of nutrients to the plants (Fitter, 1991; Johnson et al., 1997; Landis and Fraser, 2008). In our study, also a shift in AMF response from mutualistic to an induced parasitism in the WB containing soil substrate has to be considered (Smith and Smith, 1996; LeCroy et al., 2013). However, a very different tomato plants growth response was observed in the soil substrate containing GWB with additional AMF.

We found that the symbiotic association of AMF together with GWB had a significant positive effect on root and shoot dry weight of tomato plants even under *Fol* stress. The GWB might have influenced the plant growth by an increase in nutrient supply. Recently Prendergast-Miller et al. (2014) reported that the biochar application increased the availability of N and P to plant roots. In our study, the improved plant growth response in soil containing GWB could be due to its higher ash contents (Kloss et al., 2014). The addition of biochar alters the soil pH and in turn affects the nutrient availability to plants. Yamato et al. (2006) has shown that the application of biochar made from *Acacia magnum* increased the soil pH and productivity of maize and peanut. Li and Dong (2013) reported that the increase in soil pH from acidic to neutral resulted into healthier tomato plants. Moreover, depending on the type of feedstock, biochars made from herbaceous material are nutrient rich and release a greater amount of nutrients (Mukherjee and Zimmerman, 2013) which may be readily available to plants as compared to biochars from woody feedstock (Singh et al., 2010). The positive effects of the GWB on AMF i.e., by altering soil properties, microbial community structure (Elad et al., 2011; Quilliam et al., 2013), and an additional protection from antagonists (Warnock et al., 2007) might also have contributed to a significant increase in below ground biomass as compared to the treatment containing only compost.

Another, explanation could be that the biochar extracts are known to contain multiple organic compounds like phenols, benzoic acid, n-alkanoic acids and others (Graber et al., 2010). Many of these chemicals may pose toxic effects to plants at high concentrations and hence could trigger a mechanism known as hormesis (low dose beneficial effects but at high dose suppression of plant growth). Therefore, the consequence of hormesis may be speculated as an alternative in plant growth response under biochar application (Graber et al., 2010; Jaiswal et al., 2014). However, as both biochars utilized in this study were produced at same pyrolysis temperature, a difference in plants growth response to WB or GWB with additional AMF seems highly dependent on the type of feedstock.

We did not find any reduction in chlorophyll contents of the tomato plants with the addition of biochar in combination with compost as compared to treatment containing only compost. Whereas Akhtar et al. (2014) reported a significant reduction in chlorophyll contents due to reduction of leaf N contents in biochar treated plants. The favorable C/N ratio in the compost treated substrate infers that the nitrogen of the compost used was relatively well available to the plants (Scherer et al., 1996). The blockage of xylem vessels by fungal colonization may induce symptoms similar like water stress in plants. A *Fol* induced reduction of PSII efficiency was reported earlier by Nogués et al. (2002) and Lorenzini et al. (1997). However, our results have shown that the PSII efficiency of tomato plants was not affected by the *Fol* inoculation in biochar treated plants. This might be due to the improved water holding capacity as well as water availability to the plants in the biochar containing treatments. Asai et al. (2009) reported that the biochar application improved the hydraulic conductivity of the top soil and the xylem sap flow of the rice plants, and Haider et al. (2014) reported an increase in water use and PSII efficiency in biochar treated maize plants.

Previous studies are rather contradictory regarding the influence of biochar on AMF colonization. Solaiman et al. (2010), Makoto et al. (2010) and Rillig et al. (2010) reported an increase in AMF colonization, whereas Birk et al. (2009) and Warnock et al. (2010) reported the decrease in root colonization by AMF in the presence of biochar. The toxic effect of mineral elements or organic compounds of biochar, changes in the physicochemical properties of soil and alteration in phosphorous availability influence the AMF ability to colonize plant roots (Lehmann et al., 2011). In our study, we found a similar mycorrhization in plants treated with compost alone and in plants grown with additional WB or GWB. Interestingly, the pathogen *Fol* resulted in a higher mycorrhization in the substrate including compost or compost and WB, and in the lower mycorrhization in the compost and GWB treatment. A competition for colonization sites in plants between AMF and *Fol* is supposable as reported earlier by Cordier et al. (1998) for *Phytophthora parasitica* in mycorrhized tomato roots. That also means that both biochars had a varying effect on AMF and *Fol* and interaction thereof. Moreover, the enhanced plant growth due to the interaction of AMF and *Fol* in compost and GWB containing treatments may be attributed to an improved nutrition and tolerance to the pathogen (Borowicz, 2001). However, Estaún et al. (2010) and Steinkellner et al. (2012) reported that the AMF response depends on the host and even on the cultivar. In this study, only one tomato cultivar (Kremser Perle) was used, therefore cultivar effects have to be tested in further studies.

Our study shows that the biochar addition to the soil substrate resulted in enhanced disease incidence and severity as compared to treatment containing only compost. The increase in disease severity in the presence of biochar might be due to the sorption of plant defense or antifungal compounds on biochar surface. However, co-inoculation of *Fol* and AMF resulted in reduction of disease severity in biochar containing treatments. Dehne and Schonbeck (1979) found enhanced lignification in the endodermis due to AMF colonization suppressed Fusarium wilt in tomato. AMF colonization has a suppressive effect on soil borne diseases and the increase in root colonization ensures improved disease suppression. Earlier, Elmer and Pignatello (2011) and Matsubara et al. (2002) reported the reduction in Fusarium root rot of AMF colonized asparagus plants in the biochar amended soils. To our surprise, co-inoculation of *Fol* and AMF of tomato plants grown in compost alone (with no biochar) enhanced disease incidence and severity as compared to -AMF plants of the same treatment. The possible reasons for enhanced disease severity could be the competition between AMF and compost associated microbial community; type and quality of organic matter can also contribute to variations in disease suppression (Mandelbaum and Hadar, 1990; Ben-Yephet and Nelson, 1999).

Biochar may have either a direct effect on the *Fol* or through modification of plant response to disease stress. Biochars are known to have low level of phytotoxic compounds which can induce systemic resistance in plants and production of defense related compounds (Elad et al., 2011). In response to biotic or abiotic stresses the plant roots exude diverse array of compounds. Root exudate compounds have the ability to modulate soil microbial communities and the composition of root exudates determines the nature of plant-microbe interactions either positive or negative (Bais et al., 2006). The plants response against *Fol* through modification of root exudate compounds is plausible.

We found that the root exudates collected from different treatments had a diverse effect on *in vitro* growth and development of *Fol*. Soil amendments consisting of compost alone and/or in addition with WB or GWB resulted in significant higher microconidia germination than a compost free substrate (data not shown). This might be attributed to a nutritional effect. As reviewed by Badri and Vivanco (2009) the root growth and exudation is influenced by nutrient availability along with other biotic and abiotic factors. Previously, different nutrient deficiencies were reported to influence the level of strigolactones (Lüpez-Ráez et al., 2008; Yoneyama et al., 2012), citric acid (Neumann and Römheld, 1999), caffeic acid (Olsen et al., 1981), and malic acid (Hinsinger, 2001) in tomato root exudates. Hage-Ahmed et al. (2013a) found that the low level of chlorogenic acid in tomato plant root exudates had a suppressive effect on *Fol* microconidia germination.

Alteration in the root exudates of tomato in response to mycorrhizae has already been reported in numerous studies (Scheffknecht et al., 2006; Hage-Ahmed et al., 2013a). In our study root exudates from +AMF tomato plants from compost alone and in combination with WB treatment enhanced the microconidia germination when compared with –AMF treatments. However, the response was opposite in the root exudates from +AMF plants grown in GWB containing treatment for microconidia germination which is in contrast with the findings of Scheffknecht et al. (2006). Besides, the pattern of mycelium development may not always be in accordance with the microconidia germination rates. Interestingly, in our experiment, the lowest mycelial growth was observed in the root exudates of +AMF plants from WB containing treatment while it was at maximum level in the root exudates of +AMF plants from GWB containing treatment. These outcomes might be attributed to the effect of compost and biochar type on the composition of root exudates in addition to mycorrhizae. Earlier, Mimmo et al. (2011) reported the substrate dependent variations in the root exudation pattern in *Lupinus albus* and *Brassica napus*. The altered response could also be due to the accumulation of new compounds in root exudates which may have stimulatory or inhibitory effect on the microconidia germination and mycelial growth and development of *Fol* (Scheffknecht et al., 2006). The increase in the level of sugar contents of the root exudates has a stimulatory effect on the germination and mycelium development of *Fol* as observed by Hage-Ahmed et al. (2013a); whereas Steinkellner and Mammerler (2007) demonstrated that the low concentration of flavonoids suppressed mycelial growth of *Fol*. However, the exact mechanism and compounds involved in variable *Fol* response to root exudates taken from different soil substrate compositions and its impact on the disease development is not clearly understood. Further, studies will be required to find out the chemical differences in root exudates and their possible influence on *Fol* growth and development.

From our study it is concluded that the tomato plants growth response and *Fol* suppression with two different biochars along with AMF is dependent on the type of feedstock. Based on *in vitro* studies on *Fol* growth and development, organic soil amendments like compost and biochars has shown the ability to alter tomato root exudates and these alterations might have an essential role in determining the plant response to disease stress. Little is known about the exudate chemistry due to the target oriented metabolic profiling with respect to the detection of already specified compounds (Haichar et al., 2014). A large fraction of compounds present in root exudates is still unknown. Therefore, future research must be focused in this direction.

### Conflict of interest statement

The authors declare that the research was conducted in the absence of any commercial or financial relationships that could be construed as a potential conflict of interest.
